# The ambient air quality standards, green innovation, and urban air quality: evidence from China

**DOI:** 10.1038/s41598-023-47112-w

**Published:** 2023-11-11

**Authors:** Han Zhang, Dandan Zhang, Wenfan Qian, Shaofeng Xu

**Affiliations:** College of Quality and Standardization, Qingdao University, No. 308 Ningxia Road, Qingdao, 266071 Shandong People’s Republic of China

**Keywords:** Environmental economics, Environmental impact, Environmental economics, Socioeconomic scenarios

## Abstract

As China’s economy transitions, environmental issues have become a major concern. This study examines the impact of Ambient Air Quality Standards (AAQS) on urban air quality using panel data from 284 cities in China from 2006 to 2019. The study utilizes DID (Difference-in-Difference) models to analyze the regulatory effects of AAQS and its spatial spillover. Additionally, the serial multiple mediation models are constructed to investigate the role of green innovation. The findings reveal that the AAQS positively affects urban air quality, albeit with a notable “hysteresis effect.” Local implementation of AAQS worsens air quality in neighboring cities within a distance of 400 km, but beyond 400 km, the effect is reversed. Heterogeneity analysis shows that AAQS improves air quality in central cities, large-sized and medium-sized cities, cities with weak environmental governance, and resource-based cities. Mechanism tests suggest that AAQS may enhance urban air quality by promoting green innovation and optimizing industrial structure. Especially, either the energy-use effect or industrial-structure effect triggered by green innovation can contribute to the improvement of urban air quality.

## Introduction

Managing the balance between environmental protection and economic development is crucial for achieving sustainable development in China. The economic growth resulting from reform and opening up has propelled China to become the world`s second-largest economy. However, this rapid development has also brought about significant challenges, particularly in terms of ecological imbalance and environmental pollution, especially air pollution. Despite some improvements, the governance efforts have not met expectations. According to the 2020 China Ecological Environment Status Bulletin, approximately 40.1% of the cities at the prefecture-level and above still fail to meet the “Ambient Air Quality Standards” (National standard number: GB 3095-2012) implemented in 2012. Urban air pollution remains a persistent issue, especially during unfavorable weather conditions when severe air pollution occurrences are frequent.

**Figure 1:**
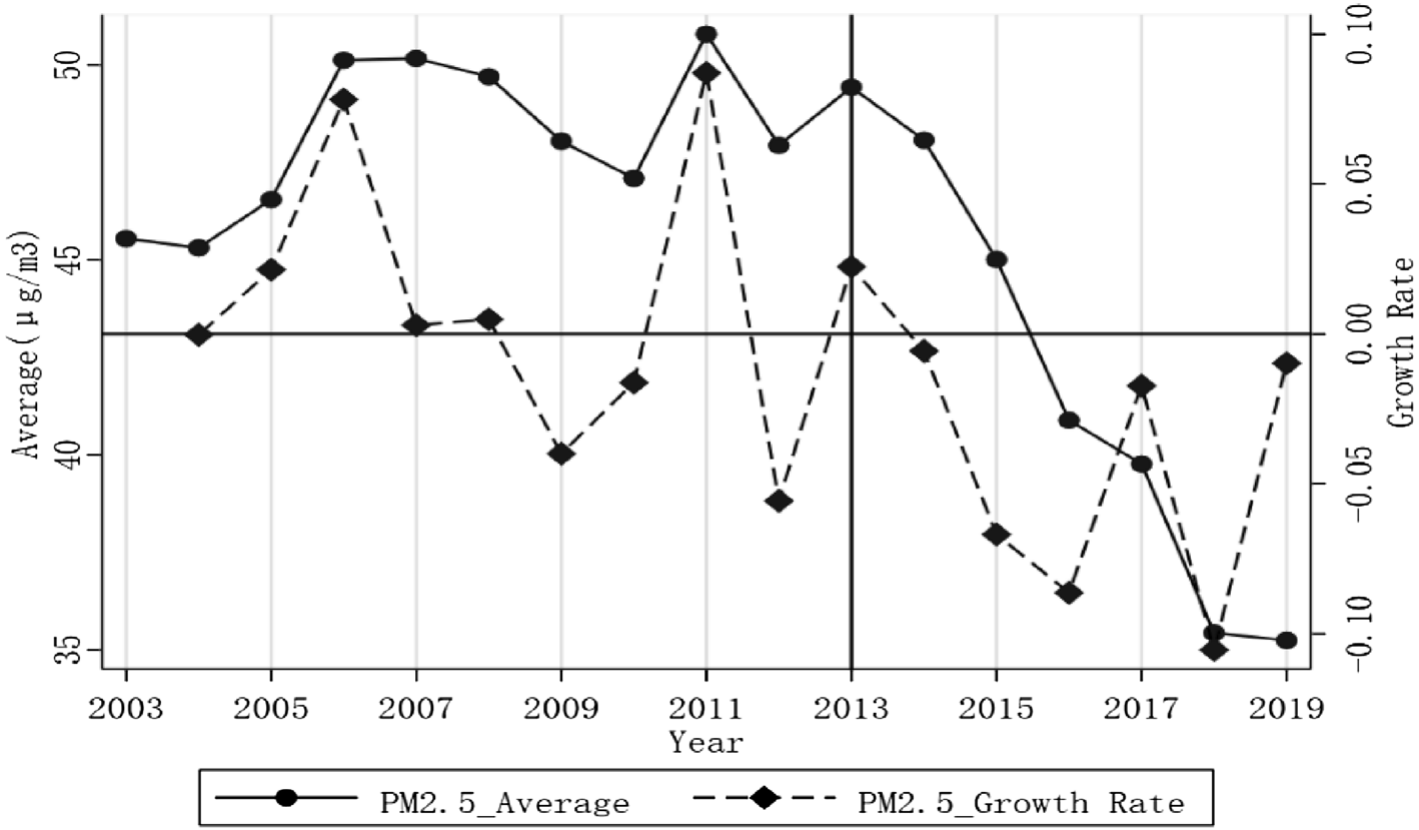
PM2.5 average value and growth rate in China from 2006 to 2019.

Figure 1 reports the annual average PM2.5 concentration and growth rate change trend in China from 2006 to 2019. It reveals that China’s annual average PM2.5 concentration has shown a significant decrease since 2013, while the annual growth rate has also changed from generally positive to negative. The preliminary analysis of the above statistics makes us wonder that the implementation of the AAQS may help to improve air quality in China.

Although the concept of “Binding” is crucial in environmental regulation, there are instances where certain environmental regulations may not necessarily improve air quality, and in some cases, they may even have the opposite effect. This phenomenon is known as the “green paradox.”^1–4^ One of the mandatory tools in environmental regulation, the AAQS, has garnered attention from the academic community since its introduction. Moreover, due to variations in research methods, samples, perspectives, and other factors, the impact of implementing the AAQS is still subject to debate. For instance, Du et al.^5^ study revealed that the establishment of automatic air quality monitoring stations as a result of the AAQS has effectively stimulated green innovation. However, these studies have focused primarily on the micro-enterprise level and have overlooked the regional air flow characteristics. Additionally, cities, as important spatial hubs for economic activities, also play a crucial role in the process of environmental regulation.

Consequently, using a panel dataset of 284 cities in China from 2006 to 2019, specifically at and above the prefectural level, we examine the impact of the AAQS on urban air quality. To avoid any potential endogenous issues, we employ the DID method, treating the implementation of the AAQS as an exogenous policy shock. The findings suggest that the AAQS, as a mandatory environmental standard, makes positive average treatment effect (ATE) on urban air quality. This study also reveals insights into the “pollution haven effect” and provides valuable information for cross-regional air pollution prevention and control efforts. Moreover, we identify two specific pathways of green innovation, through which the AAQS enhances urban air quality, both.

Our study makes several contributions to the existing literature on environmental air regulation. Firstly, unlike previous regulations, the AAQS was implemented in three stages from 2012 to 2015, covering all cities at the prefecture-level and above. This progressive and dynamic implementation allows us to explore the temporal effects and regional heterogeneity of the regulation using time-varying DID and PSM-DID models. This enhances the robustness of our results and enriches the relevant research.

Secondly, unlike previous studies, we incorporate the issue of spatial proximity or correlation into our analytical framework. This allows us to examine the general spatial spillover effects of the AAQS on air quality and investigate the heterogeneous characteristics of these spillovers at different geographical distances intervals. This provides a more objective understanding of the regulation's impact on urban areas.

Thirdly, we consider the role of green innovation as a potential mediator between the AAQS and urban air quality. By establishing a serial multiple mediation (SMM) model, we are able to identify the total and specific effects of green innovation on urban air quality, including energy-using effect and industrial-structure effect. This allows us to explore the mechanisms of green innovation in more detail and refine the existing studies.

The rest of this study is organized as follows: "Literature review" section provides a literature review, "Theoretical analysis and research hypotheses" section presents theoretical analysis and research hypotheses, "The methodology and variables" section constructs the basic and spatial DID models and provides definitions and statistical descriptions of all variables, "Empirical evidence" section presents the empirical estimation including benchmark estimation, dynamic trend test, spatial ATE test, robustness checks, heterogeneity tests, and the discussion on mediating roles of green innovation. Finally, "Conclusions, policy implications and research forecast" section presents the conclusions, policy implications, limitations, and future research directions.

## Literature review

### Environmental regulation and air quality

As a powerful tool for addressing air pollution and improving air quality, environmental regulations and policies have been extensively studied and implemented in various studies, which have largely confirmed the positive impact of these regulations on ambient air quality^6–10^. However, due to the differences in research objects, index selection or institution background, the non-positive or even complex nonlinear relationship between different environmental regulation tools and ambient air quality has been proved recently. However, recent research has revealed that the relationship between different environmental regulation tools and ambient air quality is not always straightforward and can be influenced by various factors such as research focus, choice of measurement indices, and institutional backgrounds. For instance, as the “Environmental Kuznets Curve” hypothesis^11^, some countries, particularly those in the early stages of development or with low-income economies, may prioritize economic growth over environmental concerns, resulting in the ineffectiveness of certain environmental policies^12, 13^.

Furthermore, certain environmental regulations can lead to increased production and operating costs for firms and industries, which can negatively impact their overall productivity and competitiveness in the market^14^; In order to maintain short-term profitability, some pollution-intensive plants or firms may neglect investments in research and development, equipment upgrades, and technological advancements, and instead relocate to areas with less stringent environmental regulations. This phenomenon, known as the “pollution haven effect or hypothesis” can result in the deterioration of air quality in both the original location and the new area^15–17^. Frequently, the decision of whether to comply with regulations or relocate often depends on factors such as regional differences in the intensity and enforcement of environmental regulations. It is important to note that the spatial spillover of pollution can lead to neighboring areas also experiencing a decline in air quality as a result of the relocation of polluting industries^18, 19^. In China, the asymmetric enforcement of environmental regulations between geographically adjacent areas can exacerbate this spatial self-selection effect of polluting plants^20–22^.

### Mediating roles of innovation

There are various ways in which environmental regulation and environmental quality are connected, and innovation is a key factor in addressing environmental issues through regulatory tools. On one hand, environmental regulation can incentivize or mandate polluting plants to adopt green innovation and environmentally-friendly technologies. This helps to internalize the cost of mitigating air pollution, reduce the cost of compliance with pollution regulations, and improve energy efficiency. Ultimately, this enhances the competitiveness of businesses in the market, which is known as the “Porter Hypothesis” (PH) or the “innovation compensation effect.” On the other hand, the environmental regulation is regarded as the initial power source of innovation, and the study of the relationship between environmental regulation and innovation has garnered attention of scholars from all walks of life. Hence, it is important to consider that well-intentioned environmental technology or green innovation aimed at reducing air pollution may have unintended consequences.

#### Impact of environmental regulation on innovation

Traditionally, many scholars believe that environment regulations impose environmental compliance costs or have a negative impact on polluting firms. In other words, environment regulations reduce productivity and lower profit, which is known as “compliance costs effect”. Conversely, on a long-term and dynamic perspective, Porter and Linde^23^ challengingly points that more stringent regulations do not inevitably hinder nation`s competitive advantage, rather than often encourage companies to re-engineer their technology, stimulate innovation and enhance productivity growth to foster competitiveness, which is known as PH. Also, either empirical evidence or theoretical models offered by amounts of studies give strong support the PH^24–26^. Contrarily, both various criticisms and conflicting evidences of the PH are also seen in several literature^27, 28^. As in the relevant research in China,Fu and Jian^29^ discovered that the effectiveness of the PH relies on the implementation of well-designed regulatory standards. However, this can be challenging for many developing countries to achieve in practice. Because those nations mainly issue the strict environmental regulations, with more asymmetric information, organizational failure and serious corruption. Likewise, He et al.^30^ observed that the “strong” PH per se can hardly be supported in Chinese manufacturing enterprises currently, and anti-corruption can be a key factor to realize it.

When studying the impact of environmental regulations on green productivity or innovation, on one hand, previous studies have found environmental regulations are regarded as the initial source of green productivity or innovation^31, 32^. In evidences from China, for instance, Wang et al.^33^ pointed that within a certain level of stringency, the environmental policy benefits urban green productivity growth by inducing the “innovation offset effect.” However, Shi et al.^34^ and Feng et al.^35^ found that China`s environmental regulation can significantly inhibit innovation or green innovation efficiency. Noticeably, heterogeneous environmental regulations having a differential impact on green innovation, studies based on data from different regions and dimensions have noted either an “inverted U” or a “U” shaped between them^36–38^. Additionally, some scholars have also found a threshold effect or non-linear effect of environmental regulations on green innovation. For example, Jiang et al.^39^ demonstrated that there were synergistic effects of mandatory and non-mandatory environmental regulations on innovation and voluntary environmental management system certification also positively moderated the relationship between mandatory environmental regulations and corporate innovation.

#### Impact of innovation on air quality

The existing theoretical literature emphasizes the important endogenous role of innovation on environmental quality^40–42^. That is, innovation improves the production efficiency of enterprises, reduces energy and other factor inputs, and ultimately achieves emission and pollution reduction. The empirical literature also verifies this conclusion from multiple dimensions. For example, Costantini et al.^32^ used European industries data analysis to find that ecological or green innovations inhibit sectoral environmental degradation; Peng^43^ found that the environmental regulation in the local region promotes the green productivity, while the weighted environmental regulation in the adjacent regions behaves the opposite; Liu et al.^44^ testified that technological innovation can alleviate the aggravating effect of agglomeration on haze pollution.

Meanwhile, according to the empirical analysis of Wang and Wei^45^ showing that OECD countries with excessive technological progress, will have a rebound effect and increase CO_2_ emissions, thus the concentration of environment-related technologies for carbon reduction directly should be prior to those for energy efficiency; The autoregressive distributed-lag model estimated results of Mongo et al.^46^ indicate that, in the long-term, environmental innovations tend to lower CO_2_ emissions, whereas in the short-term the observed effect is the opposite; Liu et al.^47^ uses the data of 30 provinces in China to empirically verify that green innovation inhibits carbon emission intensity.

In summary, the current research has limitations. Firstly, there is a lack of research on the influence of China`s AAQS on air quality using model-based causal inference. Secondly, most studies have focused on provincial-level samples from China instead of city-level samples, thus neglecting the potential spatial correlation of regulation effects after implementing the AAQS. Last but not the least, there is a need for more studies to explore the potential cross-correlation or chain reaction between green innovation and other mediating variables when studying the mechanism through which ambient air regulation affects urban air quality.

## Theoretical analysis and research hypotheses

In this section, we intend to conduct a theoretical investigation to determine whether the implementation of the AAQS has a positive impact on the quality of urban air. Then, we will explore whether cities, when faced with mandatory regulations and associated costs, actively seek to reduce energy consumption or accelerate industrial transformation through green innovation in order to improve air quality. Based on this, research hypotheses are proposed.

The AAQS, implemented in three phases, has several advantages compared to other environmental laws and regulations. It serves as a national mandatory standard and command-controlled regulation tool with a shorter revision cycle and greater flexibility. This encourages non-compliant cities to focus on increased supervision and rectification. It also helps to address the information asymmetry between central and local governments, and limits cross-regional air pollution transfer. Ultimately, the implementation of the AAQS aims to prevent and control air pollution in China. However, due to the pressure of economic development and environmental protection, some 3-High enterprises may choose to relocate, and local governments may implement extreme policies that are not tailored to specific circumstances. As a result, the implementation of the AAQS not only affects local air quality, but also has an impact on neighboring regions. Since air circulation is not limited by administrative boundaries, pollution from one region can spread to adjacent areas, leading to negative spatial effect or spatially “partial benefits and overall losses.”

Furthermore, upon conducting a thorough examination of the existing body of research, we can deduce that environmental regulations compel companies to internalize the expenses associated with pollution control and reduction. As a result, there is an increase in investment in R&D, the adoption of cleaner technologies, and the promotion of green innovation. Furthermore, serving as a point of integration for environmentally-friendly development and innovation-driven approaches, green innovation has become a crucial factor in promoting a sustainable green economy at a regional level in China^48–50^. Therefore, it is plausible that the implementation of the AAQS introduces relevant policies that actively encourage innovation, particularly in areas with 3-High industrial activity. In order to avoid being adversely affected by market access barriers resulting from the AAQS, cities where the AAQS is enforced should consider adopting air pollution abatement technologies or engaging in green innovation with environmentally-friendly inputs. This approach would not only reduce tax payments for pollution but also mitigate the degradation of ambient air quality.

Given the significant impact of green innovation on environmental sustainability, here we aim to explore two effects of green innovation: “energy-using effect” and “industrial-structure effect.” The former refers to how the green innovation can improve energy efficiency, encourage the use of eco-friendly production methods, reduce fossil fuel consumption, and mitigate the negative impacts of polluting economic activities on the environment^51–53^. Yet, it is noteworthy that green innovation or clean technologies can also lead to economic growth, which may create new energy demands and increase energy consumption. This is known as the “rebound effect”, which could partially or completely offset the benefits of innovation and even contribute to environmental degradation^54, 55^.

The latter refers to that derived from implementing the AAQS, the green innovation aims to improve and enhance the environmental performance of industries. It involves upgrading and optimizing existing industries, as well as relocating and eliminating resource-based industries that have negative environmental impacts. Additionally, the green innovation focuses on cultivating or introducing environment-friendly industries. By promoting the optimization or upgradation of the industrial structure, the green innovation strives to achieve a “win–win” situation for both the environment and the economy^56–58^.

Based on the above analysis, two hypotheses are proposed as following.

### H1

The implementation of the AAQS benefits the improvement of urban air quality; meanwhile, the spatial spillover of its regulatory effectiveness cannot be ignored.

### H2

As a non-ignorable mediating factor between the AAQS and ambient air quality, the green innovation will be conducive to urban air quality by influencing energy use and industrial structure. But the direction of its influence may be uncertain in different paths.

## The methodology and variables

### Model design and variable definition

In 1982, China`s first AAQS (GB 3095–1982) was issued by the General Administration of Quality Supervision, Inspection and Quarantine of China, including the first set criteria for total suspended particles (TSP) evaluation. In 1996, the AAQS (GB 3095–1982) was substituted by the AAQS (GB 3095–1996), specifying that the concentration of PM10 and TSP must be not higher than 10 μg/m^3^ and 100 μg/m^3^. In February 2012, to better improve the ambient air quality, the Ministry of Environmental Protection of China (MEPC) and the General Administration of Quality Supervision, Inspection and Quarantine jointly released the new and stricter AAQS (GB 3095–2012) which included for the first time fine particulate matter (PM2.5) into the air quality testing system, and set up national and secondary standards, which not only reinforce the monitoring major air pollutants such as O_3_, PM10, NO_2_, and PB but also adopt more advanced measurement and updating technical to strictly test local air quality. Also, local governments are explicitly required to disclose ambient air quality data to the public and social media in real time.

Gradually, the AAQS (GB 3095–2012) was implemented in three phases, which started in 74 key cities in 2012, then continued in other 113 key cities in 2013 and lastly covered all prefecture-level cities in 2015. Based on the empirical objective of “the AAQS → urban air quality”, we follow Wang et al. and Du et al. to regard the implementation of the AAQS as a quasi-natural experiment^6, 7^ to capture its ATE on the key cities and construct a basic time-varying DID model given by following formular:

1 $$AQ_{it} = \alpha + \beta TR_{it} + \lambda \sum {X_{it} } + \mu_{i} + \delta_{t} + \varepsilon_{it}$$

where *i* = 1, ···, *N*, and *t* = 1, ··· , *T*. *N* is the cross section and *T* is the time dimensions of the panel.

The dependent variable, *AQ*, denotes the urban ambient air quality. Different from some literature, considering that before 2013, the Air Pollution Index (API) released by the MEPC covers the concentrations of six major air pollutants, including SO_2_, CO, O_3_, etc., yet does not include PM2.5. Considering that the time span of this study is between 2003 and 2019, API and AQI are not compatible with air quality measurement standards during this period. Moreover, China did not monitor and record the concentration of PM2.5 in the annual Ecological Environment Bulletin before 2012. Since implementation of the AAQS issued by the MEPC, the PM2.5 has been introduced as a major pollutant into the air quality monitoring system for the first time. In other words, as the Geng et al. and Liu et al. have done^59, 60^, PM2.5 concentration can be used as a landmark air quality indicator to test the regulatory effect. Therefore, following Han et al. and Wang et al.^61, 62^, we use the annual average concentration of PM2.5 in city *i* at year *t* to measure *AQ* of cities at prefecture level and above.

The core independent variable and dummy variable, *TR*, measures whether a city is affected by the implementation of the AAQS, which means that if city *i* belongs to treatment group (key cities) and implemented the AAQS in 2012, we will set *TR* = 1 after year 2012 (*t* = 2013,…, 2019) for the city *i*. Otherwise, the city *i* will be included in the control group, and *TR* = 0. Since all prefecture-level cities have been implemented the AAQS and there will be no cities belonging to control group after 2015, here we only estimate the impacts of the AAQS performed in 2012 and 2013; the coefficient *β* captures the ATE of implementing the AAQS on urban air quality.

The control variables, *X*, are divided into two categories, one is urban economic variables, including ①the urbanization rate (*UR*), which is proportion of non-agricultural population in total population at year-end of each city; ②the industrial sulfur dioxide emissions (*SO*_*2*_); ③industrial soot emissions (*ST*). The other category is urban weather variables, including ①the annual average temperature (*TE*);②the annual precipitation (*PR*); ③the sunshine duration (*SD*).

Other variable, *μ*_*i*_ and *δ*_*t*_ denote the city-fixed and time-fixed effects, respectively, controlling for city-level factors that do not vary with time and time-level characteristics that do not vary with city; ε_*it*_ is the residuals. To relieve potential heteroscedasticity and serial correlation, we clustered the standard errors at the city-year level.

Furtherly, considering the potential spatial spillover of the local ATE in geographical adjacent cities, we follow Jia et al. (2021) to extend model (1) by constructing a Dynamic Spatial-Dubin Difference-in-Difference (DSD-DID) model given as following formular^63^:

2 $$AQ_{it} = \xi AQ_{it - 1} + \beta TR_{it} + \rho \sum {W_{ij} \times AQ_{jt} } + \theta \sum {W_{ij} \times TR_{jt} } + \lambda \sum {X_{it} } + \mu_{i} + \delta_{t} + \varepsilon_{it}$$

where *ρ* is the spatial coefficient, representing spatial correlation of the AAQS among cities; the coefficient *ξ* measures the dynamic impact of urban air quality in the *t-1* year on that in the *t* year. If *ξ* = 0, the DSD-DID model will turn into Static Spatial-Durbin Difference-in-Difference (SSD-DID) model; the coefficient *θ* of the interaction *W* × *TR* measures the spatial spillover of the ATE; *W*_*ij*_ denotes the spatial weight matrix, which is the inverse of the spherical geographical distance between cities *i* and *j*. *W*_*ij*_ is normalized, and the diagonal element of *W*_*ij*_ is set to 0. Other variables are the same as model (1).

### Statistical description of variables

In this study, cities such as Chaohu, Bijie, Tongren, Sansha, ect, which were adjusted in administrative divisions and had a large number of missing data during the examination period, were excluded, and the final estimated sample covered 284 cities in China at the prefecture level and above from 2003 to 2019. Some missing values were filled by linear interpolation. Avoiding potential heteroscedasticity and the dependence on the regression model setting, we took all of control variables as their natural logarithm. To avoid the influence of extreme values, all variables are winsorized by 1% in the empirical analysis. All relevant data were obtained from the *China Bureau of Statistics*, *China Urban Statistical Yearbook*, *China Environmental Statistical Yearbook*, *National Oceanic and Atmospheric Administration*, and *Atmospheric Composition Analysis Group* of Dalhousie University, Canada. Table 1 gives the descriptive statistical results of variables.

**Table 1:** Descriptive statistics.

Variables	Meaning (Units)	Obs	Mean	Sd	Min	Max
*AQ*	Annual PM2.5 concentration (µg/m3)	3976	45.557	15.469	18.054	89.304
*TR*	Whether it will be affected by the AAQS (-)	3976	0.249	0.433	0.000	1.000
*ln_UR*	Logarithm of urbanization rate (-)	3976	-0.597	0.376	-1.485	0.000
*ln_SO*_*2*_	Logarithm of industrial sulfur dioxide emissions (million tons)	3976	10.218	1.185	6.563	12.380
*ln_ST*	Logarithm of industrial soot emissions (million tons)	3976	9.628	1.103	6.418	12.023
*ln_TE*	Annual averaged temperature (℃)	3976	7.419	1.028	0.000	8.043
*ln_SD*	Annual averaged sunshine duration (hours)	3976	2.630	0.463	0.531	3.165
*ln_PR*	Annual averaged recipitation (mm)	3976	6.659	0.769	3.475	7.885

## Empirical evidence

### Benchmark results

Table 2 reports the ATE of implementing the AAQS on urban air quality in line with model (1). The results of DID estimation in model (1) are shown in columns (1)–(3). Meanwhile, to avoiding sample bias due to systematic differences of urban characteristics between the treatment group and control group, the method of “Propensity Score Matching” (PSM) is used through selecting all control variables as matching covariates and then taking the cities with the closest matching score as the control group for DID estimation, which is also called PSM-DID estimation. The results of PSM-DID estimation are shown in columns (4)–(6). Columns (1) and (4) represents the result from DID and PSM-DID without control variables, respectively. Columns (2) and (5) represents the result from DID and PSM-DID only with urban economic variables, respectively.

**Table 2:** Benchmark results.

Variables	(1)	(2)	(3)	(4)	(5)	(6)
	DID	DID	DID	PSM-DID	PSM-DID	PSM-DID
*TR*	− 0.516**	− 0.881***	− 0.867***	− 0.283	− 0.612***	− 0.599***
	(− 2.230)	(− 3.770)	(− 3.727)	(− 1.221)	(− 2.665)	(− 2.616)
*ln_UR*		− 7.404***	− 7.351***		− 8.732***	− 8.514***
		(− 9.118)	(− 8.979)		(− 9.553)	(− 9.081)
*ln_SO*_*2*_		0.894***	0.887***		0.929***	0.872***
		(6.034)	(6.265)		(5.795)	(5.515)
*ln_ST*		0.261**	0.245**		0.313**	0.327***
		(2.223)	(2.150)		(2.513)	(2.666)
*ln_TE*			2.062*			5.369***
			(1.851)			(3.310)
*ln_SD*			− 1.194			− 1.121
			(− 1.515)			(− 1.466)
*ln_PR*			0.005			0.128
			(0.031)			(0.751)
*_cons*	45.685***	29.705***	33.377***	46.059***	28.549***	22.331***
	(571.148)	(20.219)	(4.729)	(565.708)	(17.942)	(2.940)
*City-Fixed Effect*	Yes	Yes	Yes	Yes	Yes	Yes
*Year-Fixed Effect*	Yes	Yes	Yes	Yes	Yes	Yes
*Obs*	3976	3976	3976	3841	3841	3841
*R*^*2*^	0.946	0.949	0.949	0.947	0.950	0.950

The t value in parentheses is calculated according to the robust standard error of clustering at the city-year level;

*, ** and ***denotes the significance levels of 10%, 5%, and 1%, respectively.

Except in column (4), the coefficients *β* of variable *TR* are significantly negative, which means that after implementing the AAQS, concentration of PM2.5 in the treatment group is significantly lower compared to the control group, indicating that the AAQS is conducive for improving urban air quality. The potential explanation is that the introduction of the AAQS created a sense of urgency for local governments and the three 3-High-polluting plants to prioritize environmental management. This led to a concerted effort to allocate resources to sectors with higher levels of green productivity. The ultimate goal was to shift the focus of economic development from a more resource-intensive approach to a more efficient and sustainable one, ultimately resulting in improved urban air quality.

Regarding the control variables, firstly, the coefficients of variable *ln_UR* in columns (2) and (4), are significant negative, reflecting that the urbanization is positive correlated with the urban air quality. Secondly, the coefficients of both variable *ln_ST* in columns (2)–(3) and (5)–(6), and variable *ln_TE* in columns (3) and (6), are significant positive, reflecting that the higher temperature, more industrial soot emissions, or more industrial sulfur dioxide emissions, the worse the air quality.

### Dynamic trend of the ATE

Passing the parallel trend test (i.e., whether there is parallel trend of air quality between the treatment group and the control group) is one of the preconditions for the DID estimation. Thus, we follow the Laporte et al.^64^ to set model (3) to explore the dynamic ATE driven from the AAQS on urban air quality by making the following formulation:

3 $$AQ_{it} = \alpha_{0} + \sum\limits_{j = - 5}^{6} {\gamma_{t} \cdot TR_{{i\;\;t_{0} + j}} } + \lambda \sum {X_{it} } + \mu_{i} + \delta_{t} + \varepsilon_{it}$$

where *TR*_*ito*+*j*_ is dummy variable, representing that take the value of 1 for the city *i* has been implemented the AAQS for *j* years since node year *t*_*0*_ and 0 otherwise. The value of *j* ranges from − 5 to − 1and from 1 to 6, denoting the period from 5 to 1 year before and from one year to five years after the implementation of the AAQS, respectively. The coefficient *γ* estimated the ATE of the AAQS in the *j*th year before and after its implementation. If the significance of coefficient *γ* passes statistical significance test, there will be a statistically difference in air quality between the treatment group and control group in the *j*th year, before or after implementing the AAQS. That is to say, the AAQS has a significantly improve urban air quality. All control variables in model (3) are same as those in model (1).

**Figure 2:**
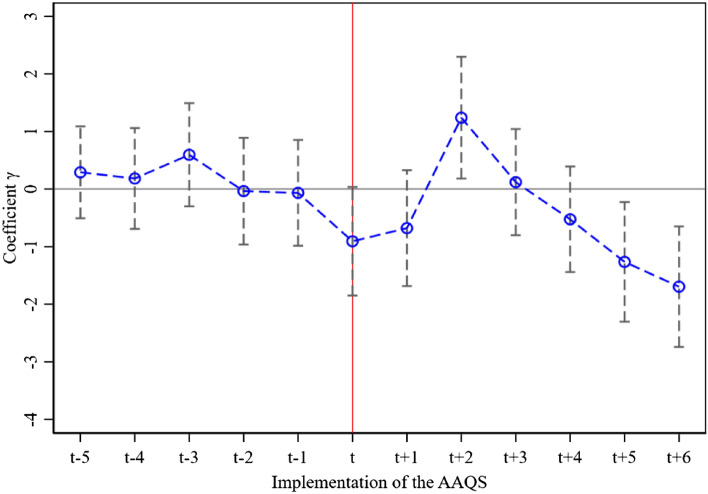
The dynamic ATE of the AAQS on urban air quality.

Figure 2 reports that before the implementation of the AAQS (*j* < 0), the coefficient *γ* did not pass the significance test, reflecting that there is no significantly gap of air quality between the treatment group and the control group; that is, the AAQS make no significant impact on urban air quality. After the implementation of the AAQS, the coefficient *γ* increases in first 2 years and then begin to continuously decreases in last 4 years, but only showing significantly positive and negative, in the second year (*j* = 2) and in the 5th and 6th year (*j* = 5 and 6), respectively.

The results manifests that there is an inverted “V-shaped” trend in the ATE of the AAQS on urban air quality; that is in the early implementation of the AAQS, for the most of 3-High polluting enterprises, there possibly is path dependence of “crude” production mode and take time to achieve an innovation-driven “green” mode, because as a command-and-control type of environmental regulation tool, the AAQS is more of an external administrative type of intervention for highly polluting or energy-consuming plants, even if they opt for R&D investment in green technologies; however, green innovation is a long-term, high-risk process, and it is difficult to achieve a complete transformation of traditional production patterns in the short term. Plus, the information asymmetry caused by collusion between local governments and plants. Hence, a lagging ATE of the AAQS on urban air quality occurs, which is also called “hysteresis effect”.

### Spatial spillover of the ATE

#### General spatial spillover test

Columns (1) and (2) of Table 3 report the regression results for SSD-DID model and columns (3) and (4) for DSD-DID model. All coefficients *β* of the variable *TR* are negative significantly, which basically verifies that the AAQS still significantly improves urban air quality even after considering spatial correlation of urban air quality. Also, the spatial coefficients *ρ* of the interaction *W* × *AQ* are significantly positive in all columns, indicating that the air quality among cities has a significant positive spatial effect of “One takes on the color of one`s company.” Specifically, the joint prevention and control of air pollution and collaborative management of cities in close geographical proximity is required to improve urban air quality. Notably, all the coefficients *θ* of the interaction *W* × *TR* displays that the ATE of local AAQS makes a significantly positive impact on concentration of PM2.5 of geographically neighboring cities. In other words, there is negative spatial spillover of the ATE driven by local AAQS on air quality of neighboring cities. This might owe to the transregional migration of polluting enterprises or the lack of cross-regional joint prevention and control mechanism, reflecting the occurrence of “pollution haven effect”.

**Table 3:** Test results of spatial spillovers.

Variables	(1)	(2)	(3)	(4)
	SSD-DID	SSD-DID	DSD-DID	DSD-DID
*TR*	− 0.368**	− 0.346*	− 0.501***	− 0.471**
	(− 1.966)	(− 1.851)	(− 2.727)	(− 2.574)
*W* × *TR*	0.878***	0.922***	8.212***	5.417***
	(2.973)	(2.861)	(25.323)	(16.207)
*L1.AQ*			0.292***	0.307***
			(24.569)	(25.220)
*Spatial ρ*	0.976***	0.963***	1.125***	1.131***
	(176.500)	(114.491)	(81.095)	(71.105)
*Control variables*	Yes	Yes	Yes	Yes
*City-Fixed Effect*	Yes	Yes	Yes	Yes
*Year-Fixed Effect*	Yes	Yes	Yes	Yes
*Obs*	3976	3976	3692	3692
*R*^*2*^	0.052	0.047	0.134	0.134

The t value in parentheses is calculated according to the robust standard error of clustering at the city-year level.

*, **, and ***denotes the significance levels of 10%, 5%, and 1%, respectively.

#### Heterogeneous spatial spillover test

Considering that the spatial spillovers or spatial ATE of the AAQS may have potentially heterogeneous regulatory effects on air quality of neighboring cities with different geographical distance. To evaluate heterogeneous spatial ATE, we refer to Sigman^65^ to make following model:

4 $$AQ_{it} = \theta \sum\limits_{\varphi } {\sum {W_{ij}^{\varphi \sim \varphi + 100} \times TR_{jt} } } + \beta TR_{it} + \rho \sum {W_{ij} \times AQ_{jt} } + \lambda \sum {X_{it} } + \mu_{i} + \delta_{t} + \varepsilon_{it}$$

where *W*_*ij*_^*φ*~*φ*+100^ denotes the spatial weight matrix with range [*φ*, *φ* + 100] km, *φ* with 100 km as the geographical distance interval, taking the values 0, 100,…,500, respectively. When the distance is within the range, *W*_*ij*_ takes the reciprocals of geographical distance between cities *i* and *j*, otherwise it is 0. The remaining variables have the same meaning as the model (2).

**Figure 3:**
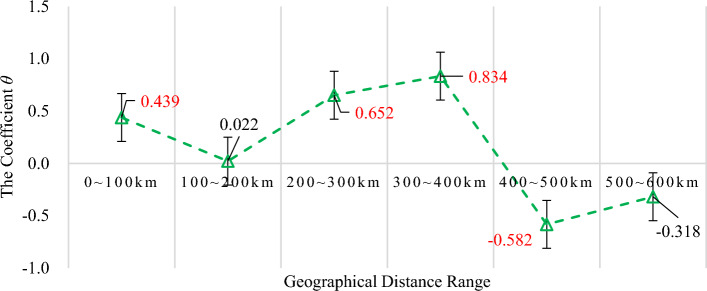
Heterogeneous spatial ATE of the AAQS.* Note*: The values of coefficients *θ* marked in red indicate that they have passed a significance statistical test.

Figure 3 reports the heterogeneous spatial spillovers of the ATE based on the estimated value of the coefficient *θ* in model (4) with six geographical distance intervals. It reveals that the impact of implementing the AAQS on air quality in neighboring cities differs significantly. The effects can be characterized as a roughly “W-shaped” trend, revealing an initial decrease, followed by an increase, and then a gradual leveling off. Within a 400 km distance, there is a “pollution haven effect” or “beggar-thy-neighbor effect”, for neighboring cities. This may result from that location choice and migration distance of heavily polluting enterprises are the result of a balance between migration costs among different regions, local environmental compliance costs, and expected production returns. The implementation of AAQS in China undoubtedly strengthens the local ambient air regulation costs. While it forces some highly polluting enterprises to transform and develop green technologies, it also compels some heavily polluting enterprises to relocate or transfer, hence negative spatial ATE of implementing the AAQS on air quality.

Moreover, the greater the geographical distance, the worse the spatial ATE on air quality. Particularly, cities located 300–400 km away from the key cities experience noticeable negative consequences. In other words, within 400 km, the negative spatial ATE on air quality of China`s AAQS shows an increasing trend as the distance increases. The substantial disparities in spatial ATE of AAQS on urban air quality within a 400 km radius may be attributed to the significant differences in economic development levels among different regions in China, which roughly show a spatial pattern of decreasing strength from east to west, and the gradient characteristics of environmental regulation strength also show a weakening trend from east to west.

Nevertheless, the situation is reversed for cities located 400–600 km away, especially those within the 400–500 km range. It is likely that the implementation of local AAQS has led to the spillover of green technology or demonstration effects in these neighboring areas, known as the “pollution halo effect”. Thus, so far *H1* has been basically testified.

### Robustness checks

#### Placebo test

The DID estimation is subject to the premise that there is no significant difference in air quality trends between treatment group and control group prior to the implementation of the AAQS and, therefore, to construct a virtual exogenous shock to the implementation of the AAQS. Following Chetty et al. and Cai et al.^66, 67^, we conduct a placebo test, that is, randomly select one year as the implement timing of the AAQS for new treatment group comprised of randomly selected cities from sample. To increase the power of the placebo test, the random generation process is repeated 800 times in this study.

**Figure 4:**
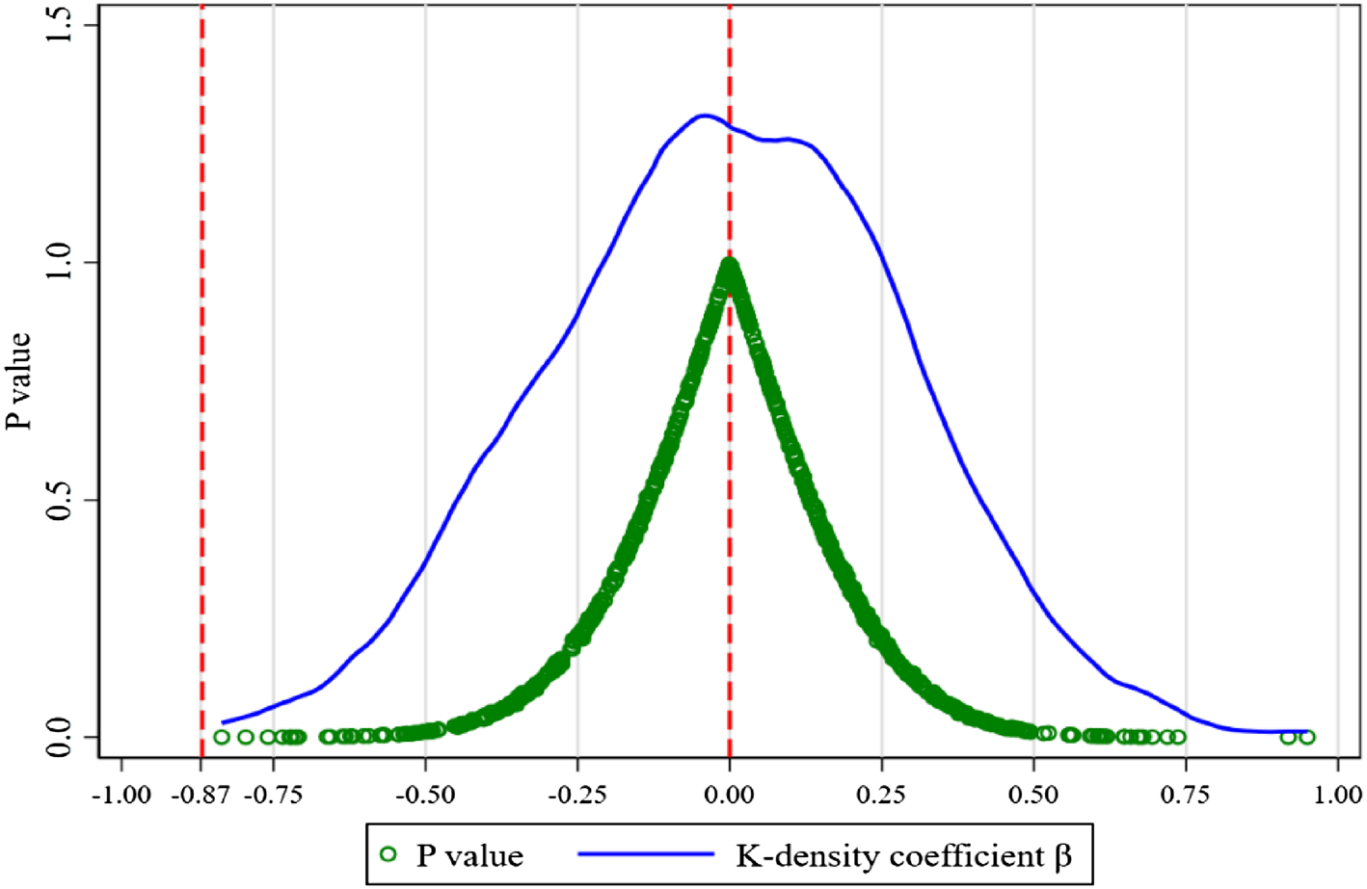
The placebo test.* Notes*: X axis represents the coefficients *β* of the variable TR from 800 random estimations of placebo test; Y axis represents the P values and K-density coefficients from each random regression. The gray solid line is the kernel density distribution curve of all 800 estimated coefficients, whereas the gray dots are corresponding P values. The left red dashed line is − 0.867, the value of coefficients *β* in columns (3) of Table 2 and the right one denotes the zero.

Plotted in Fig. 4, the kernel density (K-density) estimated coefficients *β* of the variable *TR* with corresponding *P* values are normally distributed around 0 and completely independent of the *β* value shown in Table 2. It indicates that the mean ATE value (i.e., − 0.454) of implementing the AAQS is significantly totally different from the placebo test results (i.e., 0), which exclude the differences in the ATE of environmental regulation between the treatment group and control group caused by other random factors, reflecting the robustness and credibility of the benchmark results in Table 2.

#### IV estimation

Considering that cities implementing the AAQS may also be affected by other potential factors that interfere with the accuracy of the benchmark results, we follow Hering and Poncet and Chen et al.^68, 69^ to choose the ventilation coefficient (*VC*) of cities as an instrumental variable (IV) for whether a city is included among the key cities, which is negatively correlated with the probability to be selected as a treatment group or key city. Because the higher the level of ventilation is in a certain city, the lower the concentration of PM2.5 or higher air quality that can be regulated, satisfying the correlation assumption of effective instrumental variables.

The formula of ventilation coefficient is: *VC*_*it*_ = *WS*_*it*_ × *BLH*_*it*_, where *VC*, *WS*, and *BLH* represents the average ventilation coefficient wind speed, and atmospheric boundary layer height, respectively. The original data for *WS* and *BLH* were obtained from the latitude and longitude raster meteorological data released by t*he European Centre for Medium-Range Weather Forecasts (ECMWF).* We used ArcGIS to parse them into the data of 284 prefecture-level and above cities in China from 2006 to 2019*.* Also, the logarithmic form of the variable *VC* is used in the DID regression.

Hence, the term *ln_VC* × *PT* interacted the logarithm of *VC* with the time dummy variable (*PT*) for the implementation of the AAQS is equivalent to the variable *TR*. Seen from column (1) of Table 4, the coefficient of the interaction *ln_VC* × *PT* is still significantly negative as same as that of *TR*, which means that after eliminating the endogeneity in selecting of treatment group cities, the implementation of the AAQS is still effective in improving urban air quality.

**Table 4:** Robustness check results.

Variables	(1)	(2)	(3)	(4)	(5)	(6)
	IV estimation	The AQ is represented by the maximum PM2.5	The AQ is represented by the minimum PM2.5	Deleting sample of municipalities, provincial capitals and sub-provincial cities	Eliminating the interference of the low-carbon pilot policy	Eliminating the interference of the expectation
*TR*		− 1.343***	− 0.454**	− 0.842***	− 0.821***	− 1.014***
		(− 4.531)	(− 2.362)	(− 3.489)	(− 3.657)	(− 2.646)
*ln_IV* × *PT*	− 0.098***					
	(− 3.225)					
*LC* × *T*					− 0.104***	
					(− 2.884)	
*P1.TR*						0.129
						(0.267)
*P2.TR*						0.402
						(0.951)
*_cons*	24.158***	37.958***	17.801***	23.594***	77.067***	16.531**
	(3.347)	(5.455)	(3.000)	(3.396)	(3.914)	(2.360)
*Control variables*	Yes	Yes	Yes	Yes	Yes	Yes
*City-Fixed Effect*	Yes	Yes	Yes	Yes	Yes	Yes
*Year-Fixed Effect*	Yes	Yes	Yes	Yes	Yes	Yes
*Obs*	3976	3976	3976	3472	3976	3408
*R*^*2*^	0.951	0.937	0.947	0.952	0.951	0.950

The t value in parentheses is calculated according to the robust standard error of clustering at the city-year level.

*, **, and *** denotes the significance levels of 10%, 5%, and 1%, respectively.

#### Other robustness tests

First, reported in columns (2) and (3) of Table 4, to substitute the mean of PM2.5 concentration, either the maximum or minimum value of PM2.5 concentration was used to represent dependent variable, *AQ*, to test based on model (1). Shown in columns (2) and (3) of Table 4, The negative value of the coefficients *β* are both consistent with results of the benchmark regression in Table 2.

Next, the DID regression is performed after excluding the samples of municipalities directly under the central government, provincial capitals, and sub-provincial cities. According to the report in column (4) of Table 4, the coefficient *β* is still significantly negative.

Again, in 2010, the National Development and Reform Commission issued the policies to carry out “Pilot Projects for Low-carbon cities” (PPLC), identifying the first batch of low-carbon piloting in five provinces (such as Guangdong, Liaoning, Hubei, etc.) and eight cities (such as Tianjin, Chongqing, Shenzhen, etc.). Hence, the interaction term *LC* × *T* of the dummy variable measuring whether the PPLC has been implemented (*LC*) and the time trend variable (*T*) for the low-carbon pilot cities is added to in the model (1), aiming to eliminate the impact of the PPLC on urban air quality. Similarly, the coefficient *β* in column (5) of Table 4 is still significantly negative albeit the significantly negative coefficient of the interaction *LC* × *T*, which is lower coefficient *β*, reflecting that the AAQS is the dominant policy improving urban air quality.

Lastly, we examined the possible impact of expected effects on the urban air quality, for the reason that different cities might have different expectations and preparations process for the implementation of the AAQS, which may cause some bias to the estimation results. To avoid the impact of such expectation, we added the dummy variables *P1*.*TR* and *P2*.*TR* for whether a city was affected by the AAQS previous one and two years its implementation into the model (2). Still, the coefficient *β* in column (6) of Table 4 remains significantly negative and the coefficients of *P1*.*TR* and *P2*.*TR* are not statistically significant, demonstrating that the expectations did not affect the ATE of AAQS on urban air quality.

Totally, the above results of robustness check furtherly and fully prove the robustness of the DID benchmark results.

### Reginal heterogeneity tests of ATE

Knowing that the AAQS can have a driving effect on urban air quality in the full sample, yet the regions of China have distinctly different characteristics in terms of economic development, population distribution, resource endowment, and facing different environmental constraints, varying the effectiveness of the environmental regulations on air quality in China^5, 7, 70^. Therefore, this section further verifies the ATE of implementing the AAQS on the regional heterogeneity across four aspects: economic zone, population size, environmental governance, and resource constraint (see Table 5).

**Table 5:** Regional heterogeneity tests results.

Variables	Economic zone				Population size			Environmental governance		Resources constraints	
	(1)	(2)	(3)	(4)	(5)	(6)	(7)	(8)	(9)	(10)	(11)
	East Zone	Central Zone	West Zone	Northeast Zone	Large-sized	Medium-sized	Small-sized	Strong-intensity	Weak-intensity	Non-resource-based cities	Resources -based cities
*TR*	− 0.260	− 0.896**	0.046	0.549	− 0.742*	− 0.800***	− 0.698	− 0.116	− 1.338***	− 0.431	− 0.870**
	(− 0.709)	(− 2.029)	(0.081)	(1.395)	(− 1.935)	(− 2.803)	(− 0.611)	(− 0.352)	(− 4.558)	(− 1.514)	(− 2.201)
*_cons*	120.081***	66.420***	120.408***	21.151**	34.158***	44.636***	28.219***	82.977***	8.202	33.554***	16.452*
	(6.731)	(4.139)	(6.487)	(2.255)	(3.210)	(4.831)	(4.343)	(8.552)	(1.228)	(4.259)	(1.818)
*Control variables*	Yes	Yes	Yes	Yes	Yes	Yes	Yes	Yes	Yes	Yes	Yes
*City-Fixed Effect*	Yes	Yes	Yes	Yes	Yes	Yes	Yes	Yes	Yes	Yes	Yes
*Year-Fixed Effect*	Yes	Yes	Yes	Yes	Yes	Yes	Yes	Yes	Yes	Yes	Yes
*Obs*	1204	1120	476	1162	1428	2394	154	2114	1862	2422	1554
*R*^*2*^	0.973	0.947	0.916	0.928	0.955	0.942	0.915	0.951	0.952	0.951	0.953

The t value in parentheses is calculated according to the robust standard error of clustering at the city-year level.

*, **, and *** denotes the significance levels of 10%, 5%, and 1%, respectively.

#### Heterogeneity of different economic zone

Based on the China`s economic zones that every city located, this section divides the overall samples into four sub-samples including eastern cities, western cities, central cities and northeast cities to further investigate whether the heterogeneous ATEs of the AAQS exist. The coefficients *β* of columns (1)–(4) in Table 5 show that the implementation of the AAQS has a significant improvement in air quality only for the central cities.

Possible causes include that: On the one hand, since the central zone is rich in resources and generally dominated by secondary and traditional industries, such as steel industries, machinery industries, industries chemical industries, coal industries and so forth, the implementation of the AAQS is considered not only an opportunity to accelerate the transformation and upgradation of smokestack industries, but also a government assessment pressure to effectively transfer or eliminate backward production capacity. Thus, relative to other cities, the AAQS more significantly improves air quality of central cities.

On the other hand, as the western zone is a relatively backward economy with weak industrial base, coupled with the weaker intensity of environmental supervision there, air pollution environmental problems are not prominent. Additionally, the eastern zone is mainly dominated by labor-intensive or tertiary industries, and the original air quality is relatively high; therefore, the regulatory effect of implementing the AAQS is hardly apparent. Plus, for the northeast zone, although the proportion of heavy industries as central zone, its concept of environmental protection is relatively conservative, and the production mode is easy to fall into the “path dependence;” Hence, the effectiveness of AAQS faces relatively evident resistance.

#### Heterogeneity of different city scale

Based on the population size, the overall samples are divided into three sub-samples: large-sized cities (population > 5 million), medium-sized cities (1 million < population ≤ 5 million), and small-sized cities (population ≤ 1 million). The coefficients *β* of columns (5)–(7) in Table 5 display that the implementation of the AAQS has a positive effect on air quality in both large-sized and medium-sized cities. Because by comparison, large-sized and medium-sized cities have more complete industrial categories and systems, industrial transformation faces lower costs of “technology inertia”, and the transformation of production patterns from pollution and sloppiness to green intensification is more efficient Conversely, small-sized cities have a single industrial structure and face higher degree of industrial isomorphism, resulting in the “scope diseconomies” for industrial transformation or upgrading. Therefore, the boost to air quality of small-sized cities from the implementation of the AAQS is not evident.

#### Heterogeneity of different environmental governance

Based on the environmental governance intensity, the overall samples are divided into two sub-samples: strong-intensity cities and weak-intensity cities. Here following the Zor and Zhang et al.^71, 72^, we calculate the frequency of keywords, such as “environmental protection” “air” “green” “low carbon” “PM2.5” and other words related to ambient air regulation, in local government work reports of each city from 2006 to 2019, and then use the sum of all frequency values to measure intensity of environmental regulation. If the value of a city is higher than the mean value of the whole sample, then the city will be considered as a strong-intensity one that attaches more importance to or emphasis on environmental governance and make more pollution control efforts. Otherwise, the city will be taken as a weak-intensity one.

The coefficients *β* of columns (8) and (9) in Table 5 display that the implementation of the AAQS has a positive impact on air quality of weak-intensity cities and insignificant negative effect on the strong-intensity cities, which infers that the AAQS gives heavy polluting industries located in weak-intensity cities greater pressure to face, therefore, the increasing marginal utility of regulation effect can be effectively brought into play.

#### Heterogeneity of different resources constraints

According to the “National Plan for Sustainable Development of Resource-based Cities (2013–2020)”(State Council Gazette Issue No. 35 Serial No. 1466) issued by the State Council of China in 2013, 262 resource-based cities were identified^73^. Knowing that different resource constraints will also affect the impact of the environmental regulation^74–76^, the overall samples are divided into two sub-samples: non-resource-based cities and resource-based cities.

Columns (10)–(11) in Table 5 reveal that the implementation of the AAQS failed to exert a significant positive effect on air quality of non-resource-based cities, while the stronger effect on air quality improvement was observed in resource-based cities. The possible reason for this is that, with a higher abundance of energy resources and under the environmental pressure induced by the AAQS, resource-based cities have more serious “race to the top.” Thus, the majority of 3-High plants are required to improve their environmental technology. As a result, urban pollution emissions have decreased, leading to increased investment in research and development of green technology. This investment aims to enhance green innovation and develop new energy sources to combat urban air pollution. In contrast, non-resource-based cities experience lower environmental pressure. Consequently, their environmental governance is less active or fails to fully enforce environmental regulations, resulting in the inefficiency of the AAQS in addressing their air pollution.

### Mechanism discussion: the mediating role of green innovation

The previous results indicate that the implementation of the AAQS has had a significant positive impact on urban air quality. As a result, the question remains as to how exactly these improvements are being achieved. In this study, we aim to highlight and examine the role of green innovation, which is triggered by the implementation of the AAQS and subsequently leads to changes in urban air quality.

**Figure 5:**
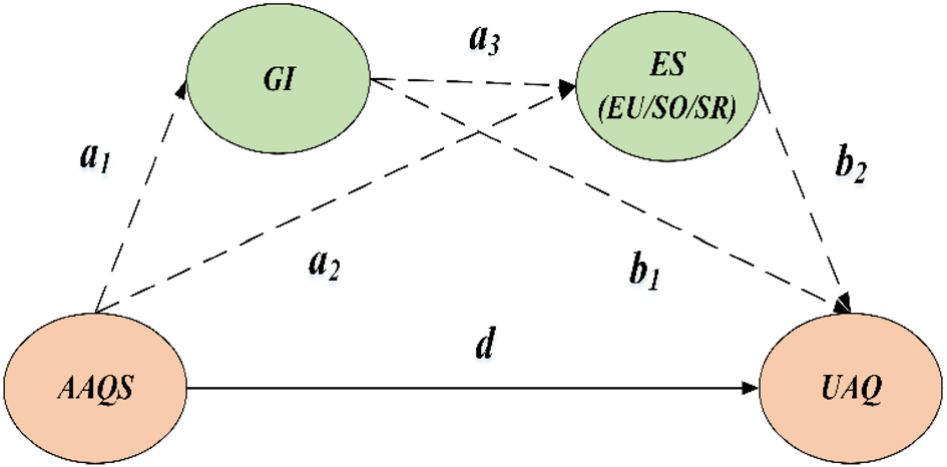
The diagram of SMM model.

To testify the *H2*, we construct a serial multiple mediation model to identify and compare different mediating paths of green innovation, through which the AAQS affects the urban air quality (see Fig. 5), with the specific equation as follows:

5 $$GI_{it} = a_{0} { + }a_{1} TR_{it} + \lambda \sum {X_{it} } + \mu_{i} + \delta_{t} + \varepsilon_{it}$$

6 $$ES_{it} = a_{0} + a_{2} TR_{it} { + }a_{3} GI_{it} + \lambda \sum {X_{it} } + \mu_{i} + \delta_{t} + \varepsilon_{it}$$

7 $$AQ_{it} = a_{0} + dTR_{it} + b_{1} GI_{it} + b_{2} ES_{it} + \lambda \sum {X_{it} } + \mu_{i} + \delta_{t} + \varepsilon_{it}$$

where the dependent variable in model (5) or the independent variable in model (7), *GI*, denotes the green innovation, which is measure by the number of green patent applications. The relevant data of green patents granted is from *Chinese Research Data Services* (*CNRDS*) Platform. In the SMM model estimation, to eliminate the right-skewed distribution problem, the value of *GI* is characterized by adding 1 to the number of green patents granted and then taking the natural logarithm.

The dependent variable in model (6) and the independent variable in model (7), *ES* including the two types of channel variables driven by green innovation. The one is the energy-using, *EU*, which is expressed by value of annual energy use or consumption and is measured by the value of standard coal consumption converted by total natural gas (i.e., coal gas, natural gas) supply, liquefied petroleum gas supply, and electricity consumption. The relevant data of *EU* is from *China Urban Statistical Yearbook*. In the SMM model estimation.

The other is the industrial structure, including the rationalization of the industrial structure, *SR* and the optimization of the industrial structure, *SO*. The former, *SR*, is measured by the Thiel index or Theil’s entropy measure, the formula of which is $SR = \sum\limits_{i = 1}^{3} {\left( {y_{i} } \right)\ln \left( {{{y_{i} } \mathord{\left/ {\vphantom {{y_{i} } {l_{i} }}} \right. \kern-0pt} {l_{i} }}} \right)}$, where *y*_*i*_ means the proportion of the added values of the primary (*i* = 1), secondary (*i* = 2) or tertiary (*i* = 3) industries in GDP, and *l*_*i*_ means the proportion of the employment of the primary (*i* = 1), secondary (*i* = 2) or tertiary (*i* = 3) industries in the aggregate employment; The latter, *SO*, is calculated by the following steps. Firstly, the ratio of value added of the primary, secondary or tertiary industries to GDP is considered a component in the vector, thus getting a three-dimensional vectors *X*_*0*_ = (*x*_*1,0*_, *x*_*2,0*_, *x*_*3,0*_). Then, calculate the angle *θ*_*j*_ (*j* = 1, 2, 3) of each industry between vectors *X*_*0*_ and the vectors *X*_*1*_ = (1, 0, 0), *X*_*2*_ = (0, 1, 0), *X*_*3*_ = (0, 0, 1) arranged from low to high, as following formula: $\theta_{j} = \arccos \left( {\frac{{\sum\limits_{i = 1}^{3} {\left( {x_{i,j} \cdot x_{i,0} } \right)} }}{{\sum\limits_{i = 1}^{3} {\left( {x_{{_{i,j} }}^{2} } \right)^{1/2} \cdot } \sum\limits_{i = 1}^{3} {\left( {x_{{_{i,0} }}^{2} } \right)^{1/2} } }}} \right)$. Finally, we get the value of *SO* according to the formula $SO = \sum\limits_{k = 1}^{3} {\sum\limits_{i = 1}^{k} {\theta_{j} } }$. The relevant data of *SR* and *SO* is from *China Urban Statistical Yearbook*.

Also, *SR* is used to measure the adaptation of industrial structure the changes in science and technology, consumption demand structure, basic quality of the population and resource conditions, reflecting reasonable allocation of production factors and the coordination among industries. Then, *SO* refers to the dynamic process of gradual changes in the industrial structure depending on the law of economic development, reflecting the evolution of industrial structure from elementary stage to advanced stage.

According to models (5)–(7) and shown in Fig. 5, the direct effect (DE) of the AAQS on urban air quality (UAQ) is* d*; the total mediating effect, TME = *a*_*1*_ × *b*_*1*_ + *a*_*2*_ × *b*_*2*_ + *a*_*1*_ × *a*_*3*_ × *b*_*2*_, where *a*_*1*_ × *b*_*1*_, *a*_*2*_ × *b*_*2*_ or *a*_*1*_ × *a*_*3*_ × *b*_*2*_ is called specific mediating effect (SME). Thus, the total effect, TE = DE + ME = *d* + *a*_*1*_ × *b*_*1*_ + *a*_*2*_ × *b*_*2*_ + *a*_*1*_ × *a*_*3*_ × *b*_*2*_. Obviously, there are mainly three mediating paths (A, B, C) through which implementation of the AAQS influences UAQ. In particular, the path A (*AAQS → GI → UAQ*), evaluated by the equation* a*_*1*_ × *b*_*1*_, indicating how the *GI* per se mediate the relationship between the AAQS and UAQ. The path B (*AAQS → ES → UAQ*), evaluated by the equation* a*_*2*_ × *b*_*2*_, indicating how the *ES*(*EU/SR/SO*) per se mediate the relationship between the AAQS and UAQ. The path C (*AAQS → GI → ES → UAQ*), evaluated by the equation* a*_*1*_ × *a*_*3*_ × *b*_*2*_, indicating how the relationship between the *AAQS* and* UAQ* is mediated by *GI* acting on either *EU* (i.e., the “energy-using effect”) or *SO/SR* (i.e., the “industrial-structure effect”).

Table 6 reports the estimation results of the mediating roles of green innovation based on SMM model (5)–(7) with the help of bootstrap method. Regarding the one specific pathway (Path B) of action under the *ES* (i.e., energy use or industrial structure), two specific pathways (Path A and Path C) of action under the *GI* (i.e., green innovation) perspective, we observed the following:

**Table 6:** Mechanism test results.

Path	*AAQS → GI*	*AAQS → (GI) → EU → UAQ*		*AAQS → (GI) → SO → UAQ*			*AAQS → (GI) → SR → UAQ*		
Independent variables	Dependent variables								
	(1) *GI*	(2) *EU*	(3) *AQ*	(4) *SO*		(5) *AQ*	(6) *SR*		(7) *AQ*
*TR*	1.361***	0.130	− 0.749**	0.022***		− 0.876**	− 0.101		− 0.797**
	(10.558)	(1.302)	(− 2.089)	(5.007)		(− 2.453)	(− 1.139)		(− 2.208)
*GI*		0.224***	− 0.472***	0.014***		− 0.462***	− 0.182***		− 0.482***
		(4.350)	(− 3.191)	(10.030)		(− 3.180)	(− 3.670)		(− 3.224)
*ES(EU/SO/SR)*			− 0.420			− 33.558***			0.297*
			(− 1.477)			(− 4.487)			(1.701)
*_cons*	− 5.919***	3.205***	52.946***	1.849***		112.198***	− 3.693***		49.003***
	(− 6.877)	(3.456)	(4.663)	(44.826)		(6.613)	(− 5.052)		(5.720)
*Control variables*	Yes	Yes	Yes	Yes		Yes	Yes		Yes
*City-Fixed Effect*	Yes	Yes	Yes	Yes		Yes	Yes		Yes
*Year-Fixed Effect*	Yes	Yes	Yes	Yes		Yes	Yes		Yes
*Obs*	3976	3976	3976	3976		3976	3976		3976
SME	***a***_***1***_** × *b***_***1***_	***a***_***2***_** × *b***_***2***_	***a***_***1***_** × *a***_***3***_** × *b***_***2***_	***a***_***1***_** × *b***_***1***_	***a***_***2***_** × *b***_***2***_	***a***_***1***_** × *a***_***3***_** × *b***_***2***_	***a***_***1***_** × *b***_***1***_	***a***_***2***_** × *b***_***2***_	***a***_***1***_** × *a***_***3***_** × *b***_***2***_
	− 0.643*** (− 2.806)	− 0.055 (− 0.865)	− 0.128** (− 2.183)	− 0.629*** (− 2.698)	− 0.739*** (− 8.275)	-0.649*** (-8.789)	-0.657*** (-2.763)	-0.030 (-1.269)	-0.074** (-1.987)
DE	− 0.749** (− 2.074)			− 0.876** (− 2.074)			− 0.797** (− 2.043)		
TE	− 1.574** (− 2.140)			− 2.893*** (− 5.875)			− 1.557** (− 2.262)		

The t value in parentheses of all variables and z value of SME, DE, and TE of SMM in parentheses are calculated according to the robust standard error of clustering at the city-year level.

*, **, and *** denotes the significance levels of 10%, 5%, and 1%, respectively. The value of interaction term *a*_1_×*b*_1_, *a*_2_×*b*_2_, and *a*_1_×*a*_3_×*b*_2_ denotes the SME level of Path A (*AAQS→GI→UAQ*), Path B (*AAQS→EU/SO/SR→UAQ*), and Path C (*AAQS→GI→EU/SO/SR→UAQ*), respectively.

#### The Path A analysis: the SME of the GI per se

The coefficients *d* of the variable *TR* in the columns (3), (5), and (7), as the value of direct effect (DE) of the AAQS on urban air quality, are all significantly negative, which again supports the robustness of the benchmark results in table (2). Meanwhile, the coefficient *a*_1_ of the variable *TR* in the column (1) is significant positive, indicating that the implementation of the AAQS is helpful to green innovation of prefectural-level and above cities and adding new evidence to the “PH.” Yet, the coefficients *b*_*1*_ of the variable *GI* in the columns (3), (5) and (7) are all negative with significance, indicating the growth of green innovation may reduce the urban air quality. Thus, all the specific mediating effects (SME) of green innovation (*a*_*1*_ × *b*_*1*_) are significant negative, reflecting that considering green innovation as a mediating factor, the impact of the AAQS on urban air quality is unchanged and keeps positive, which means that Path A (*AAQS → GI → UAQ*) is significantly established.

#### The Path B analysis: the SME of the ES per se

On the one hand, the coefficients *a*_*2*_ of the variable *TR* in the columns (6) is significantly positive, indicating that the implementation of the AAQS promote the optimization of industrial structure; while the coefficients *a*_*2*_ in the columns (2) and (4) are not statistically significant. That is, the implementation of the AAQS does not directly affect energy use and rationalization of industrial structure. On the other hand, the coefficient *b*_*2*_ of the variable *EU*, *SR* and *SO* in the columns (3), (5) and (7) are all negative at the significance level of 1%, implying that energy use and industrial structure are both negatively correlated with urban air quality.

Totally, the SME values, *a*_*2*_ × *b*_*2*_, of only the *SO* between *AAQS* and *UAQ* is statistically negative, while that of either *EU* or *SR* does not reach statistical significance. This illustrates the implementation of the AAQS ultimately improves urban air quality by improving the optimization of industrial structure. In other words, the Path B only under the optimization of industrial structure per se (*AAQS → SO → UAQ*) is verified. The possible reason is that as an external administrative means, the AAQS per se hardly influence the urban energy consumption without technological breakthroughs or innovative upgrades, and its consequent “pollution haven effects” may forcibly change the industrial distribution, rather than based on spontaneous market forces and the law of economic evolution, therefore hindering its impact on the energy use and rationalization of industrial structure.

#### The Path C1 analysis: the energy-using effect of the GI

The coefficient *a*_*3*_ of *GI* in the column (2) is significantly positive (0.224) at the significance level of 1%, indicating that the higher the level of green innovation, the more energy is consumed, providing evidence that there might be a “rebound effect.” Accurately, calculated by the bootstrap method, the resulting value *a*_*1*_ × *a*_*3*_ × *b*_*2*_ of SME of green innovation through energy use in column (3) is negative (− 0.128) at the significance level of 5%, which also means the proportion of SME of the green innovation through energy use in the TE (− 1.574) is 8.13%. In other words, the implementation of the AAQS strongly promotes the green innovation, furtherly intensifies energy consumption, yet the green technology leads to the fewer pollutant emission, and finally the improves urban air quality. Consequently, the Path C1 (*AAQS → GI → EU → UAQ*) is sensitive. In short, as an intermediary channel between the AAQS and UAQ, the green innovation plays obvious role through the “energy-using effect.”

#### The Path C2 analysis: the industrial-structure effect of the GI

The coefficients *a*_*3*_ of the *GI* in columns (4) and (6) is significantly positive and negative, respectively, indicating that on the one side, the green innovation promotes the optimization of urban industrial structure; yet, on the other side, impedes the rationalization of industrial structure, which is not line with our expectation, but still consistent with some researchers findings that when faced with mandatory environmental policy or extreme “one-size-fits-all”, the regulated plants have to reallocate original R&D resources and transfer them to green innovation, resulting in “crowding-out effect” of green innovation, even resource mismatch, and breaking the inherent coordination between different industries^77, 78^. Other scholars have found empirically that there is threshold effect of environmental regulations on green transition of the industrial economy or green innovation^79^. When exceeds a certain the level of regulations intensity, the AAQS could induce enterprises to innovate and thus adjusts the industrial structure toward a rationalization level, while when its intensity is relatively low, it may be detrimental to the rationalization of industrial structure.

Moreover, the coefficients *b*_*2*_ of the *SO* and *SR* in columns (5) and (7) is significantly negative and positive, respectively, indicating that optimization of industrial structure helps to improve urban air quality while the rationalization of industrial structure appears to represent an opposite trend. Precisely, calculated by the bootstrap method, the corresponding value (*a*_*1*_ × *a*_*3*_ × *b*_*2*_) of SME of green innovation through optimization and rationalization of industrial structure, are negative (i.e., − 0.649 and − 0.074) with the significance at level of 1% and 5%, respectively, which also express the proportion of SME of the green innovation through optimization and rationalization of industrial structure in the TE (− 1.574) is 21.74% and 4.75%, respectively.

To wit, the implementation of the AAQS strongly promotes the green innovation, furtherly either upgrades industrial structure or restrains the industrial collaboration, yet indirectly both improving the urban air quality. Therefore, like Path C1, both Path C2.1 (*AAQS → GI → SO → UAQ*) and Path C2.2 (*AAQS → GI → SR → UAQ*) are also proved to be effective. In short, as an intermediary channel between the AAQS and UAQ, compare to its “energy-using effect”, green innovation plays stronger “industrial-structure effect.” Hence, so far *H2* has been mostly testified.

## Conclusions, policy implications and research forecast

### Conclusions

With the Chinese economy in a period of transition, there is increasing concern about environmental issues in political and academic circles. The implementation of the AAQS has become an important tool for regulating the environment and promoting the disclosure of local air quality information. Recent academic research has focused on evaluating the effectiveness of these standards.

This study examines the impact of the AAQS on urban air quality using panel data from 284 cities in China from 2006 to 2019. The DID model is used to analyze whether the implementation of the AAQS leads to improvements in air quality. The results show that the AAQS significantly reduces concentrations of PM2.5 in cities that implement the standards. This conclusion holds even after conducting various robustness checks.

The study also explores the spatial spillover effects of the AAQS on air quality using the Spatial-Dubin DID model. The results reveal a “W-shaped” trend, where local cities implementing the AAQS have a negative impact on air quality of neighboring cities within a distance of 400 km, the evidence of “pollution haven effect”, but a positive impact on cities 400–600 km away, the evidence of a “pollution halo effect.”

Furthermore, heterogeneity tests show that the implementation of the AAQS has a positive impact on air quality in cities located in the central region, large-sized and medium-sized cities, cities with weak environmental governance, and resource-based cities. Noteworthily, mechanism analysis suggests that either green innovation per se or industrial structure optimization per se acts as a positive mediator between environmental regulations and urban air quality. Additionally, we also find that the AAQS can influence energy use or industrial structure through green innovation, ultimately resulting in enhancement of urban air quality by stimulating green innovation, influencing energy use and industrial structure optimization.

### Policy implications

This paper empirically analyzes regulation effect of the environmental mandatory standards with its spatial spillover and heterogeneity performance from meso dimension of prefecture-level and above city in China, the developing countries in economic transition, and digs deeper into acting mechanism behind it based on the mediating roles of green innovation. Combining with the main findings of this study, the three relevant policy recommendations are proposed as following for further improving urban air quality and promoting green transformation from a global perspective.

First, in order to effectively curb the issues on “pollution haven effect” or “pollution transfers” from developed regions or countries, and improve the overall ambient air quality, the developing countries should also consider the spatial spillover of regulatory effects and formulate strategies and system for joint prevention and control of trans-regional and transnational air pollution, both among different cities within their own country and with geographically neighboring countries. Also, strengthening the assessment, supervision and accountability of environmental governance, alongside engaged citizens pushing for change.

Second, responding to the importance of issuing the national mandatory standards for environmental regulation in China, with its heterogeneous regulatory effects on cities of various sorts, it is necessary for the developing countries in transition to implement command-based or mandatory environmental regulatory tools in order to achieve both economic growth and environmental quality or effective environmental management. Additionally, to achieve the ambient goals in a gradual and orderly manner, countries should develop targeted and differentiated urban environmental regulatory strategies based on their own regional development, urban scale, resource endowments, and environmental constraints. For example, for large-sized cities, it is sensible to adopt environmental regulations that combines government compulsion type with market incentive type; for resource-based cities, it is advisable to accelerate green reform to get rid of the established resource over-dependence, upgrade the old industries, and seize opportunities to fully unleash potential of green production.

Third, in regards to the impacts of green innovation, specifically “energy-using effect” and “industrial-structure effect,” driven by the implementation of environmental air regulations, there are two main aspects to consider. On the one hand, it is important to introduce fiscal incentives or subsidy policies to stimulate green research and development (R&D) as well as the importation of green technologies. This will help in fully developing renewable energy technologies and improving the efficiency of energy utilization. On the other hand, there is a need to strengthen the inducing effect of green innovation on industrial transformation and upgrading. This can be achieved by building an industrial structure system that is driven by green innovation. Through this approach, a new path can be formed by promoting industrial upgrading and collaboration among different industries. Ultimately, these efforts will have positive effects on the overall ambient air quality.

### Research forecast

In various stages of implementing the AAQS to enhance urban air quality, the approaches and mechanisms involved differ. This study focuses solely on the role of green innovation as a mediating variable, without analyzing other significant mechanisms like resource allocation, interactions between central and local governments, and FDI. Therefore, future research should explore and examine these aspects. Also, due to limitations in quantitative methods and data availability, the selection and measurement of relevant control variables in this study may not be sufficiently comprehensive. Hence, it is important to measure these variables more scientifically and carefully. Additionally, it should be noted that the division of geographical intervals here is not in accordance with a strict standard, and the geographical distance boundary of the spatial spillover of ATE on air quality cannot be accurately estimated by spatial DID model that we built. We will further explore this in the follow-up study.

## Data Availability

The datasets used and/or analyzed during the current study are available from the corresponding author upon reasonable request.
